# Interstitial pneumonia via the oropharyngeal route of infection with *Encephalitozoon cuniculi*

**DOI:** 10.1371/journal.pntd.0012130

**Published:** 2025-09-08

**Authors:** Mariana Souza Santos, Rodrigo A. da Silva, Lucas Nunes de Souza, Nicolli R. Victorino, Rayane C.B. Pereira, José Guilherme Xavier, Ronalda da Silva Araújo, Elizabeth C. Pérez, Anuska M. Alvares-Saraiva, Maria Anete Lallo

**Affiliations:** 1 Programa de Patologia Ambiental e Experimental, Universidade Paulista (UNIP), São Paulo, Brasil; 2 CETESB-Companhia Ambiental do Estado de São Paulo, São Paulo, Brasil; Universidade do Estado do Rio de Janeiro, BRAZIL

## Abstract

Microsporidia causes opportunistic infections in immunosuppressed individuals. Mammals shed these spores of fungi in feces, urine, or respiratory secretions, which could contaminate water and food, thereby reaching the human body and causing infection. The oral route is the most common route of infection, although experiments have demonstrated that intraperitoneal and intravenous routes may also spread infection. Respiratory tract infection, although considered to be possible, has not been reported to date. The present study, therefore, aimed to demonstrate infection with the microsporidia of *Encephalitozoon cuniculi* via the oropharyngeal route as a model for opportunistic pneumonia. The objectives were the study of opportunistic pneumonia in general while also confirming transmission via the respiratory route to expand the understanding of the epidemiology of zoonoses. C57BL mice, both male and female, up to 12 weeks of age, and free of specific pathogens (SPF) were inoculated (Infected group) or not infected (Uninfected group) with 1 × 10^7^ spores of *E. cuniculi* via the oropharyngeal route. The animals immunosuppressed with cyclophosphamide (Cy) were infected (Cy-Infected group) or not infected (Cy-Uninfected group), and then assessed for the influence of immunosuppression on infection. In both groups, animals inoculated with 0.9% saline solution via the oropharyngeal route served as controls (Sham and Cy-Sham groups, respectively). After 14 days of infection, the lungs of all animals were retrieved for histopathological analysis, phenotyping of lung inflammatory cells using flow cytometry, measurement of fungal load using qPCR, and measurement of the serum levels of Th1, Th2, and Th17 cytokines. The results revealed that the infected animals developed interstitial pneumonia characterized by perialveolar inflammatory infiltrative lesions with a predominance of lymphocytes and plasma cells. However, the fungal load and the extent of the inflammatory infiltrate were relatively lower in the Cy-Infected group, with a predominance of the CD8^+^ T lymphocyte population in the lungs compared to Infected group. In the Infected group not treated with Cy, an increase in the population of alveolar macrophages (F4/80^+^CD11b^-^SiglecF^+^) was noted, along with higher fungal load and inflammatory infiltrate in the lungs, indicating a further pronounced *Encephalitozoon* pneumonia compared to the immunosuppressed animals. The infected groups presented Th1 cytokine profiles, with the Cy-Infected group exhibiting increased Th17 levels. Collectively, these results demonstrated that oropharyngeal infection promoted pneumonia caused by *E. cuniculi* in mice treated or not with cyclophosphamide, with greater severity occurring in the non-immunosuppressed mice, thereby establishing this model as a suitable one for interstitial pneumonia via aspiration.

## Author summary

Opportunistic fungi infection in immunosuppressed individuals may lead to pneumonia that is lethal. The airways are the most common routes for these infections. The opportunistic fungal species *Encephalitozoon cuniculi* is associated with pneumonia, although this species has not been reported to cause airway infection to date. The present study aimed to demonstrate that the oropharyngeal route, which is a frequent route of infection in patients with reflux, could lead to severe pneumonia in the mice without immunosuppression associated with a low CD8^+^ T lymphocyte response.

## Introdution

Microsporidia are obligate intracellular parasitic fungi classified within the phylum *Microsporidia*. They produce spores ranging in size from 1 to 12 µm, characterized by a thick spore wall and a coiled polar tubule—features that are hallmarks of this phylum [1]. These primitive eukaryotic organisms lack mitochondria and are known to cause opportunistic infections, typically establishing within hosts with compromised immune systems [2]. In mammals, microsporidiosis is caused by 17 recognized species, although more than 1,700 species have been identified within the phylum. The most clinically relevant species include *Enterocytozoon bieneusi*, *Encephalitozoon intestinalis*, *Encephalitozoon cuniculi*, and *Encephalitozoon hellem* [2,3]. Infections are commonly intestinal or systemic, and their pathogenicity varies depending on the microsporidian species involved and the host’s immune status [4].

Opportunistic respiratory infections are critical determinants of patient prognosis and represent major contributors to prolonged hospital stays and significantly increased mortality in immunocompromised individuals [5]. Microorganisms can reach the lower respiratory tract through four primary routes: (1) aspiration of oropharyngeal contents; (2) inhalation of contaminated aerosols; (3) direct extension from adjacent infected regions; and (4) hematogenous dissemination from extrapulmonary sites of infection, such as the gastrointestinal tract. Among these, aspiration and hematogenous spread are particularly noteworthy. One study reported that aspiration of microorganisms from the upper airways during sleep occurs in approximately 45% of healthy individuals and in up to 70% of those with impaired protective reflexes—such as patients with alcohol dependence, drug use, epilepsy, or under sedation [6]. The lungs are the principal target of these infections, regardless of the etiological agent. However, immunosuppressed hosts are especially vulnerable to developing clinical pneumonia [5].

Microsporidian spores might be acquired by ingestion, inhalation, direct contact with the conjunctiva, contact with animals, or individual-to-individual transmission, ultimately causing infection [2]. In infections with microsporidia have been conducted experimentally in mice, and other rodents via oral, hematogenous, or intraperitoneal routes. In *E. cuniculi*, these routes of infection reportedly caused a disseminated disease with respiratory compromise, particularly in immunocompromised individuals [3,7]. A study reporting the signs of symptomatic respiratory infection by *E. cuniculi* in kidney transplant recipients concluded that lifelong immunosuppression increases the risk of infection with respiratory microsporidiosis in these patients [8]. Moreover, while the cases of *E. cuniculi* pneumonia in immunocompromised patients, particularly the transplant recipients, are reported, infections with the respiratory route have not been elucidated to date. In this context, the present study aimed to evaluate infection with *E. cuniculi* via the oropharyngeal route in mice immunosuppressed with cyclophosphamide or those non-immunocompromised, as a model of infection caused by aspiration of oropharyngeal contents.

## Methods

### Ethics statement

All experimental procedures were in accordance with the Brazilian law on the use of experimental animals, safety, and use of pathogenic agents. The study was approved by the Ethics Committee for Animals, Universidade Paulista (approval number: 1528190220, 04/02/2020).

### Animals

Specific-pathogen free (SPF) C57BL/6 male and female mice (n = 30), each 10–12-week-old, were procured from the “Centro de Desenvolvimento de Modelos Experimentais para Biologia e Medicina” at the Federal University of São Paulo, Brazil. The animals were housed in microisolators with controlled temperature (22–24 °C) and humidity (45% to 55%) under SPF conditions and a 12 h light and 12 h dark photocycle, throughout the experimental period, at the Animal Facility of Paulista University. The animals were provided with irradiation-sterilized pelleted food and autoclave-sterilized water ad libitum.

### *E. cuniculi* spores

*E. cuniculi* (genotype I) spores were purchased from Waterborne Inc. (New Orleans, LA, USA). The spores were grown in a rabbit kidney cell line (RK-13, ATCC CCL-37) using Roswell Park Memorial Institute (RPMI) medium supplemented with 10% fetal calf serum (FCS) and gentamicin at 37 °C under 5% CO_2_ atmosphere. The spores were then harvested from the tissue culture supernatants, washed thrice in phosphate-buffered saline (PBS), and finally counted using a hemocytometer.

### Cyclophosphamide treatment

Mice in the immunosuppressed group were treated with 200 mg/kg Cy via intraperitoneal injection once a week (Genuxal, Asta Medica Oncologia, São Paulo, Brazil). This treatment was begun 14 days prior to pathogen inoculation followed by a weekly administration throughout the experimental period [9].

### Oropharyngeal inoculation in mice

The mice were anesthetized with sevoflurane via dispersion within a housing chamber until the animals lost movement and allowed the administration of the pathogen. Afterward, each animal was suspended, and its tongue was pulled laterally for administering the inoculum into the oropharynx using a micropipette. As an autonomous reflex action, the mouse inhaled the inoculated solution [10]. After inhalation, the tongue of the mouse was released, and the mouse was placed in a cage and monitored until it woke up from the effect of anesthesia. Later, the treatment mice were infected, through oropharyngeal aspiration, with 1 × 10^7^ spores of *E. cuniculi* dispersed in 50 µL of sterilized phosphate-buffered saline (PBS) solution. The Sham (control) group of mice, either treated or not treated with Cy, received 50 µL of sterilized 0.9% NaCl solution via the same route.

### Experimental design

**Fig 1:**
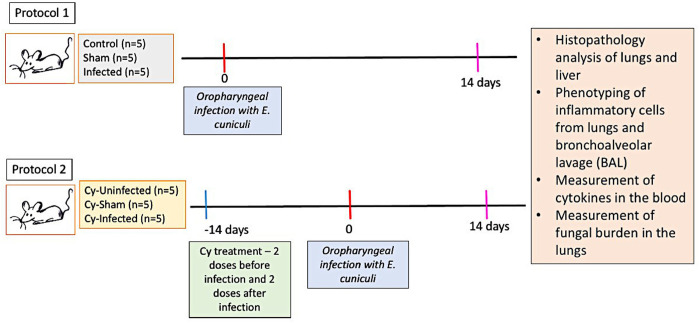
Experimental design. C57BL/6 mice were infected [or not infected, as required] with the spores of *E. cuniculi* followed by treatment [or no treatment, as required] with cyclophosphamide (Cy). After 14 days of infection, serum samples were collected to evaluate the levels of Th1, Th2, and Th17, histopathological analysis of the lungs and liver, phenotyping of the inflammatory cells in the lung, blood and bronchoalveolar lavage (BAL), measurement of the levels of cytokines in the blood, and determination of fungal burden in the lung.

The mice were subjected to two different protocols based on whether they had to be treated with Cy. Protocol 1 was applied to 3 groups of mice: Control group (n = 5) – untreated and uninfected mice; Infected group (n = 5) – mice infected with *E. cuniculi* spores via the oropharyngeal route; Sham group (n = 5) – mice that received 50 µL of sterilized the 0.9% NaCl solution. Protocol 2 was applied to the following groups: Cy-Uninfected (n = 5) – mice immunosuppressed with Cy and not inoculated with the pathogen; Cy-Infected (n = 5) – mice immunosuppressed with Cy and inoculated with the spores of *E. cuniculi*; Cy-Sham (n = 5) – mice immunosuppressed with Cy and inoculated with 50 µL the sterilized 0.9% NaCl solution. Protocol 2 was begun 14 days prior to infection (-–14 days), with the administration of a weekly dose of Cy as the immunosuppressive agent prior to the experimental infection. In the above 2 protocols, the course of infection was 14 days, after which the animals were euthanized and the tissue samples were collected for infection evaluation. The evaluations included the: histopathological analysis of the lungs and liver, phenotyping of the inflammatory cells in the lung, blood and bronchoalveolar lavage (BAL) determination, measurement of the levels of cytokines in the blood, and determination of fungal burden in the lung (Fig 1).

### Necropsy and sample collection

After applying deep anesthesia using ketamine (100 mg/kg), xylazine (2.5 mg/kg), and acepromazine (2.5 mg/kg) intraperitoneally, blood samples were collected, via cardiac puncture, in a tube containing EDTA. The tubes with blood samples were then centrifuged to obtain the plasma samples, which were stored at –80 °C until use. Afterward, the thoracic cavity of each mouse was exposed, and bronchoalveolar lavage (BAL) was performed to isolate the trachea and fix it for the infusion of 1 mL of HBSS along with 2% FBS through aspiration. This procedure was conducted twice consecutively, and the collected material was centrifuged at 2000 rpm for 5 min. Finally, the sediment containing the cells was distributed in the wells of a V-bottom 96-well plate.

In the lung tissue analysis, the lung from each mouse was retrieved and weighed, followed by the separation of the left lung lobe, which was then stored at –80 °C until used for fungal load measurements. The right lung lobe was fixed in 10% buffered formalin and then subjected to a histopathological analysis. The remaining lung lobes were mechanically dissociated (BD Medimachine, Bruino, TO, Italy), resulting in cell suspensions that were filtered through a 70 µm cell strainer and then treated with the hemolytic buffer, when necessary. The cell-containing sediments obtained in the above steps were also distributed in the wells of a V-bottom 96-well plate and subjected to phenotyping. The liver and spleen were also retrieved and fixed in 10% buffered formalin, followed by routine processing for histopathology.

### Histopathological analysis and morphometry

Paraffin-embedded tissue samples were prepared routinely for histopathology. Tissue cuts were stained with hematoxylin–eosin (H&E), analyzed, and imaged under the DM LS (Leica, German) light microscope using the DFC420 image pick-up (Leica) and Leica Application Suite version 3.1.0 image program. Histological sections of lung tissue from each animal in both the Infected and Cy-Infected groups were analyzed morphometrically. The lung perialveolar tissue area was determined from the digitized images in the morphometric analysis using the MetaMorph Microscopy Automation & Image Analysis software (Molecular Devices, California, USA). The hotspots in the pixels of the lesions of four animals from each experimental group were measured, followed by calculating the mean of each experimental group. The mean area of lesions was then determined and utilized to compare the microsporidian infection in animals between the two groups.

### Phenotypic characterization of immune cells

In order to block the Fc receptors of cells from Lung and BAL, these cells were incubated with the anti-CD16/CD32 antibody (diluted in PBS supplemented with 1% albumin-BSA) for 20 min. Afterward, the cells were washed and incubated with the following monoclonal antibodies*:* peridinin chlorophyll (PerCP)-conjugated or allophycocyanin (APC)-conjugated rat anti-mouse CD19, Phycoerithrin (PE)-conjugated rat anti-mouse CD23, Peridinin Chlorophyll Protein Complex (PerCP)-conjugated rat anti-mouse CD4, FITC-conjugated rat anti-mouse CD8, PE Cy7-conjugated rat anti-mouse F4/80, and pacific blue*-*conjugated rat anti-mouse CD11b (BD-Pharmingen, San Diego, CA). Next, the following cell phenotypes were identified in the evaluated cells: CD4^+^T cells (CD45^+^/CD4^+^), CD8^+^T cells (CD45^+^/CD8^+^), total macrophages (CD11b^+^F4/80^+^), interstitial macrophages (CD11b^+^F4/80^+^SIGLEC-F^-^), and alveolar macrophages (CD11b^-^F4/80^+^SIGLEC-F^+^). The cells were incubated at 4 °C, and after 30 min, the cells were washed, re-suspended in 200 µL of PBS, and evaluated using flow cytometry. The relevant data were acquired using BD Accuri C6 (BD Biosciences, Mountain View, CA).

### Cytokine quantification

The plasma samples were thawed and evaluated for the levels of IL-2, IL-4, IL-6, IFN-γ, TNF-α, IL-17, and IL-10 using the BD CBA Mouse Th1/Th2/Th17 Cytokine Kit (BD Biosciences, CA, USA) according to the manufactures’ instructions. Briefly, 25 µL of each sample was added and allowed to capture the beads specific to the cytokines and PE (phycoerythrin)-labeled secondary antibodies. The sample was then incubated for 2 h at room temperature in the dark. Two-color flow cytometric analysis was performed using BD Accuri C6 (BD Biosciences). The data were analyzed using FCAP Array analysis software version 3.0 (BD Biosciences, San Diego, CA, USA).

### Fungal load

The fungal load was determined by quantifying the fungal genomic DNA present in the lung tissue of each mouse using the real-time PCR (qPCR) technique. After euthanizing the mice, the lung samples were retrieved and weighed, followed by separating the left pulmonary lobe and freezing it in the RNA*later* solution (Invitroge, USA) for a maximum of 30 days. Total DNA extraction was performed using the commercial DNeasy Blood & Tissue extraction kit (Qiagen, Germany) according to the manufacturer’s instructions [11]. The extracted DNA was eluted using 100 µL of AE buffer and then stored at –20 °C until used for the qPCR analysis. The spores present in the lung tissue samples were quantified spectrometrically (BioDrop µLite, Biochrom) based on a standard curve generated based on the DNA extracted from the samples inoculated with 1 × 10^7^ purified spores/mL. Ten-fold serial dilutions of the standard, from 1 × 10^6^ to 1 × 10^1^ spores/µL, were performed in each amplification cycle (Cq) for the 18S rRNA gene of *E. cuniculi*. The best fit between the points was identified, and the coefficient of linear regression (R2) and the slope of the curve were calculated using the software. The quantitative polymerase chain reaction (qPCR) analysis was performed using the StepOne Plus Real-Time PCR (Applied Biosystems, Foster City, CA, USA). Amplification was performed in a total volume of 25 µL/reaction, which contained 12.5 µL of SsoAdvanced Universal SYBR Green Supermix 2X kit (Bio-Rad Laboratories, Inc., USA) along with 5 µL of the template DNA and the respective primers (20 pmol). Quality controls included positive DNA samples obtained from purified spores and non-template controls (NTC). The detection limit (LD) used in the present study was 10 spores/µL, the linear correlation coefficient (R2) was 0.998, and the amplification efficiency was 93.5%. The entire procedure was conducted in accordance with the Minimum Information Guide for Quantitative PCR Publication (MIQE) [12].

### Statistical analysis

T-test or variance analysis (ANOVA) was conducted followed by Tukey’s and Bonferroni’s multiple comparison post-tests for statistical analysis. All data were presented as means ±standard errors of the means (SEMs), using the significance threshold of α = 0.05 (p < 0.05). Graphs were plotted using “GraphPad Prism” version 5.0 for Windows (GraphPad Software Inc., La Jolla, CA, EUA).

## Results

### *E. cuniculi* oropharyngeal infection led to interstitial pneumonia associated with a mixed pattern of Th1, Th2, and Th17 cytokines

**Fig 2:**
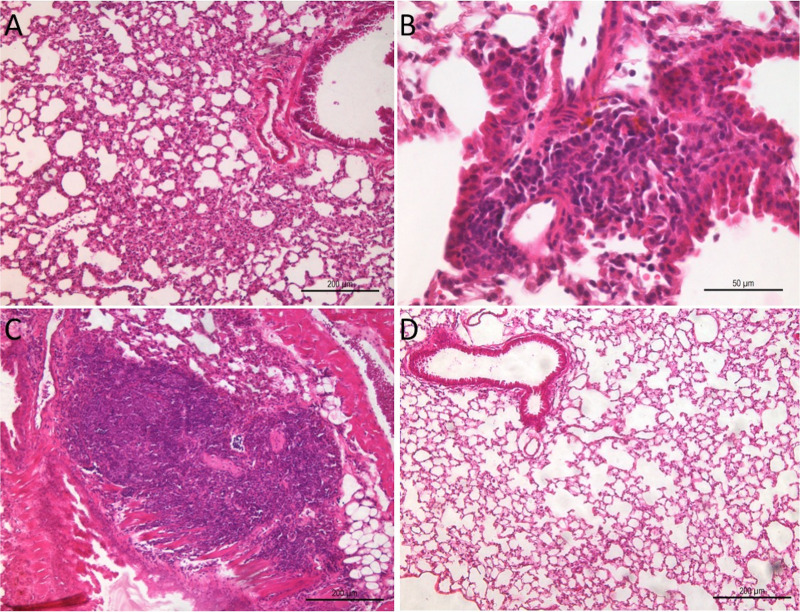
Photomicrographs of mice lungs and liver infected with *E. cuniculi* spores via the oropharyngeal route or those administered with NaCl solution via the same route (Sham group). **A)** Sham group mouse without any signs of lesions in the lung parenchyma. **B)** Infected mice lung with diffuse thickening of alveolar walls. **C)** Dense mixed inflammatory infiltrate in the lung of the Infected group mice. **D)** Lung of mice infected with *E. cuniculi* exhibiting a predominantly mononuclear infiltrate with a dense nodular appearance. (Hematoxylin and eosin [H&E] staining).

In the Sham group, the absence of lesions was predominant (Fig 2A), although eventually, the desquamation of pneumocytes was observed, which was suggestive of broncho-aspiration pneumonia. This was attributed to the administration of sterile sodium chloride solution. The animals infected with *E. cuniculi* exhibited the presence of lymphoplasmacytic inflammatory infiltrates in the alveolar parenchyma (Fig 2B and 2C). However, at times, a dense nodular mononuclear infiltrate was observed in the infected animals (Fig 2D). In the microscopic examination, the presence of *E. cuniculi* spores inside cells was observed rarely. To assess the dissemination of the infection, we performed histological evaluation of the liver, as this organ has been shown to be a target in different experimental infection routes. Nodular inflammatory infiltrates with a predominance of mononuclear cells were observed in the periportal or sinusoidal regions (zone 1) in both infected groups, which was consistent with the presence of the pathogen, suggesting the spread of the infection acquired via the oropharyngeal route (S1A and S1B Fig).

**Fig 3:**
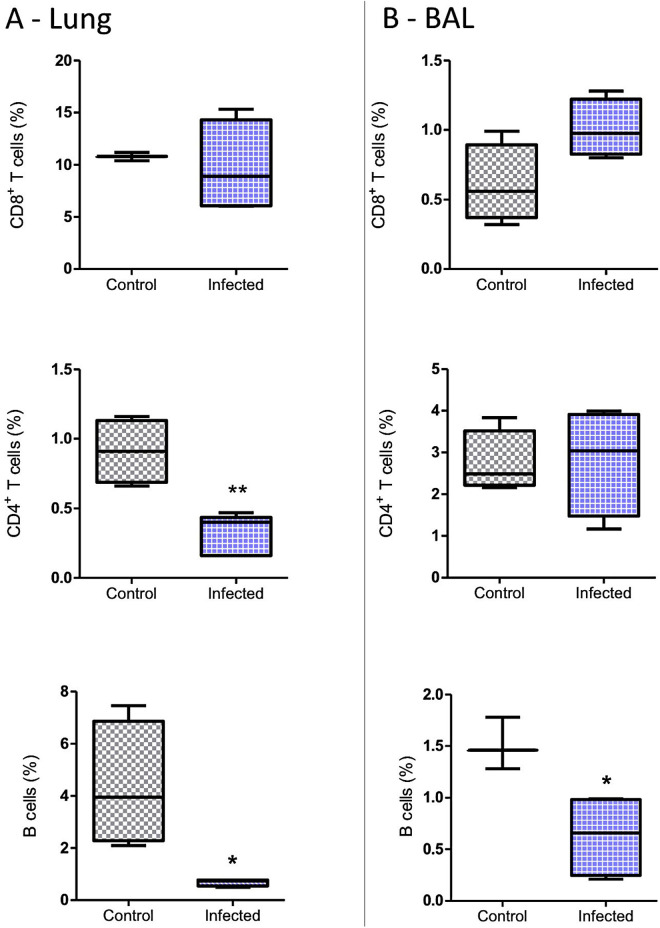
The total percentages (%) of CD8 ^+^, CD4^+^, T cell, and B cell populations in the lung (A) and BAL (B) of the mice infected (Infected group) or not infected (Control) with *E. cuniculi* spores. **(A)** Lung. **(B)** BAL. The data presented are means ±standard errors of the means (SEMs) (*p < 0.05, **p < 0.01, T-test).

No difference in the percentages of CD8^+^ T lymphocytes was noted in the lung parenchyma and BAL between the Infected group mice and control mice. However, the percentage of CD4^+^ T cells in the lungs and that of B cells in the lungs and BAL were lower in the Infected group animals compared to controls (Fig 3A and 3B). No differences in the levels of T and B lymphocytes in the blood were noted in all groups (S2A Fig).

**Fig 4:**
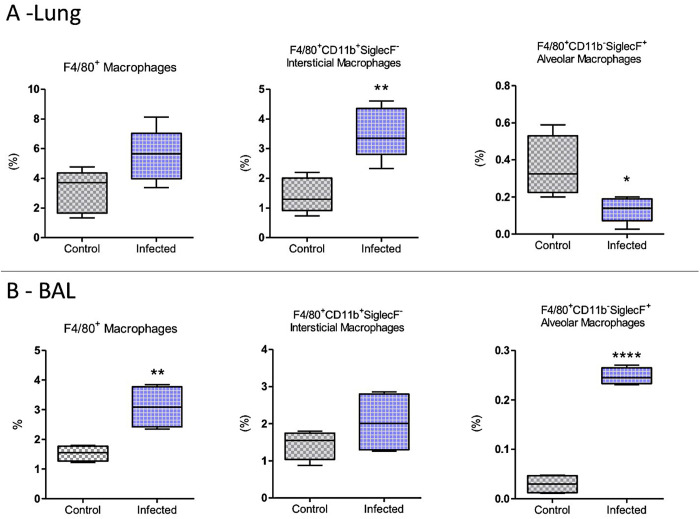
Macrophage populations in the lungs or BAL of the mice infected (Infected group) or not infected (Control group) with *E. cuniculi* spores. Percentage of total macrophages (F4/80^+^), interstitial macrophages (F4/80^+^, CD11b^+^, and SiglecF^-^), and alveolar macrophages (F4/80^+^, CD11b^-^, and SiglecF^+^) in the lungs (A) of and BAL **(B)**. The data presented are means ±standard errors of the means (SEMs) (*p < 0.05, **p < 0.01, T-test).

Further, the percentage of total macrophages was higher in the lungs of the Infected group of mice. This was attributed to the increase in interstitial macrophages (F4/80^+^CD11b^+^SiglecF^-^), while the percentage of alveolar macrophages (F4/80^+^CD11b^-^SiglecF^+^) decreased in this group (Fig 4A). In BAL, the macrophage population was increased, which was attributed particularly to the higher percentage of alveolar macrophages (F4/80^+^CD11b^-^SiglecF^+^) compared to controls (Fig 4B).

**Fig 5:**
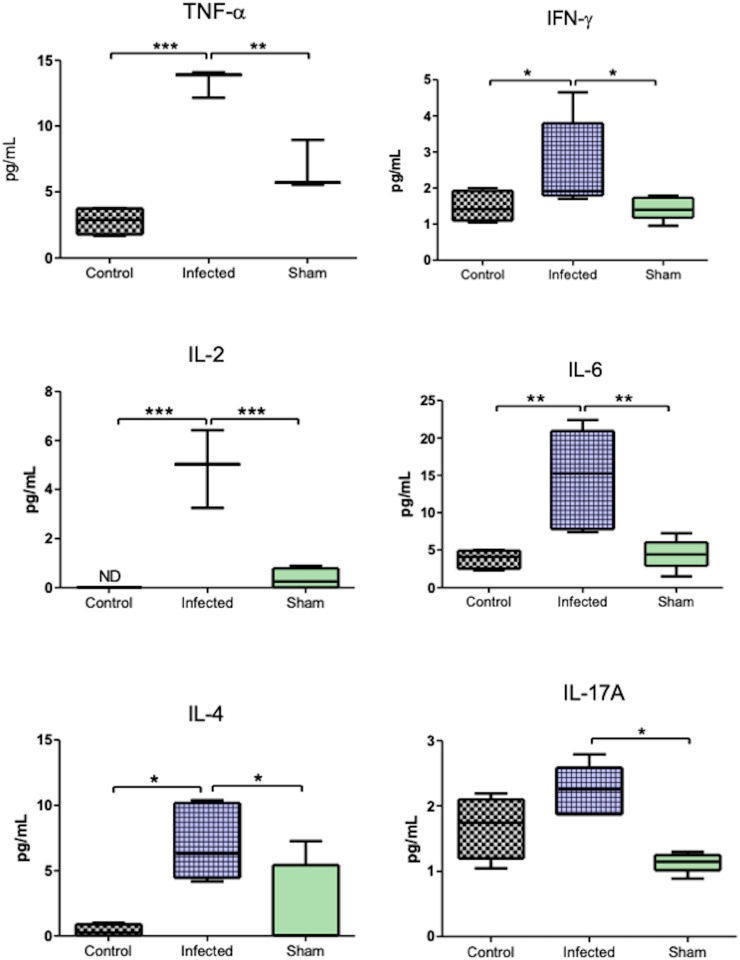
Plasma levels of TNF- α, IFN-γ, IL-6, IL-2, IL-17A, and IL-4 cytokines in the mice infected (Infected group) or not infected (Control group) with *E. cuniculi* spores and the uninfected mice that received NaCl solution via the oropharyngeal route (Sham). The data presented are means ±standard errors of the means (SEMs) [*p < 0.05, **p < 0.01, ***p < 0.001, one-way analysis of variance (ANOVA) along with multiple comparisons and Tukey’s post-tests].

*E. cuniculi* infection led to increased plasma levels of pro-inflammatory and anti-inflammatory cytokines in the Th1, Th2, and Th17 profiles compared to controls (Fig 5). In particular, levels of TNF-α and IL-6 increased 2 times, and IL-2 increased 4 times relative to controls. Infection with *E. cuniculi* also resulted in increased IFN-γ levels. Among the anti-inflammatory cytokines, IL-4 exhibited increased levels compared to those in the control mice, while IL-10 was not detected in no group. Interestingly, an increase was noted in the plasma IL-17 levels in the infected group compared to the Sham group, although no difference was noted compared to the control group (Fig 5).

### Reduction in fungal load was associated with increased CD8^+^ T lymphocyte levels in the lungs of animals immunosuppressed with Cy and infected with *E. cuniculi*

**Fig 6:**
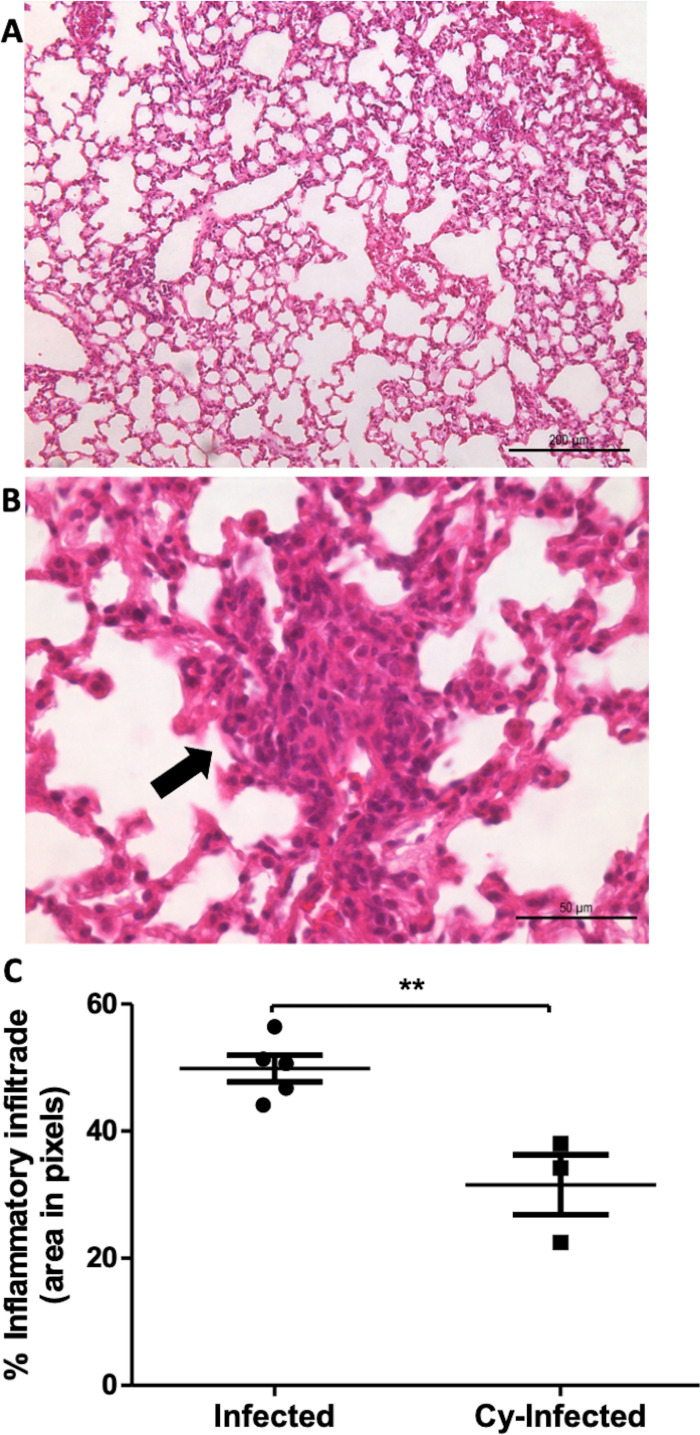
Histopathological lesions in the lungs of the mice treated with cyclophosphamide and then infected via the oropharyngeal route, and the comparative morphometric analysis of the inflammatory infiltrate in the mice infected with *E. cuniculi* and then treated [or not treated] with cyclophosphamide. **A)** Photomicrographs of the histological sections revealing discrete thickening of alveolar walls with diffuse distribution in the lungs of the mice treated with cyclophosphamide and then infected with *E. cuniculi* (Cy-Infected) **[H&E]**. **B)** Photomicrographs of the histological sections revealing a dense mononuclear infiltrate with a nodular shape in the lung of a Cy-Infected mouse **[H&E]**. **C)** Comparison of the percentage area of inflammatory infiltrate in the hotspots calculated using the metamorph software between the Infected and Cy-Infected groups. T-test revealed **p < 0.01.

In the Cy-Infected group, lymphoplasmacytic inflammatory lesions were detected in the alveolar parenchyma, similar to the Infected group (Fig 6A and 6B). However, when comparing the intensity of the lymphoplasmacytic inflammatory infiltrate by measuring the area of hotspots, the Infected group exhibited a significantly larger area compared to the Cy-Infected group (Fig 6C). This finding is generally associated with the immunosuppressive and anti-inflammatory effects of Cy.

**Fig 7:**
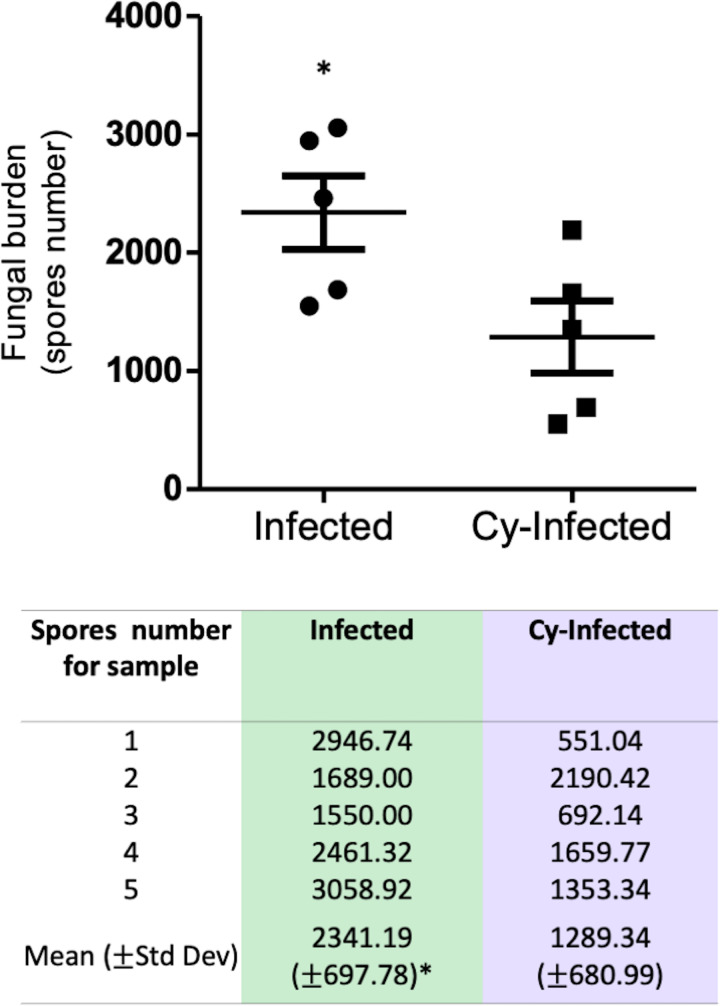
Quantification of fungal load in the lung parenchyma of the mice infected with *E. cuniculi* spores (Infected group) or immunosuppressed with cyclophosphamide (Cy) and then infected (Cy-Infected group) using the real-time PCR (qPCR) technique (Student’s *t*-test, *p* < 0.05*).

**Fig 8:**
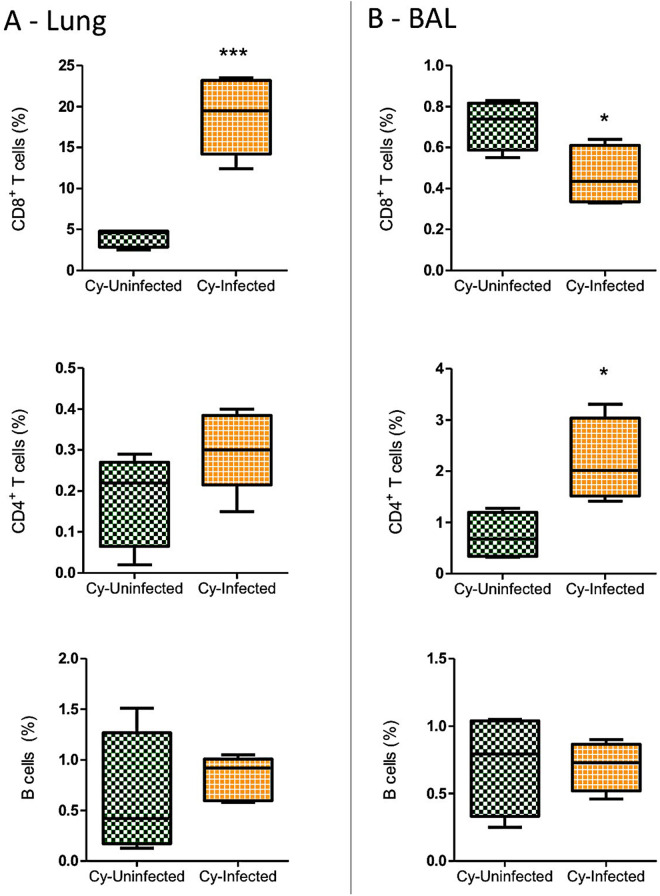
The total percentage (%) of CD8^+^ and CD4^+^ T cell and B cell populations in the lung and bronchoalveolar lavage (BAL) of the mice immunosuppressed with cyclophosphamide (Cy) and then infected (Cy-Infected group) or not infected (Cy-Uninfected group) with *E. cuniculi* spores. **(A)** Lung. **(B)** BAL. The data presented are means ±standard errors of the mean (SEMs) (*p < 0.05, **p < 0.01, T-test).

The Infected and Cy-Infected groups were compared based on the quantification of fungal load in the mouse lung using qPCR. Surprisingly, the lung fungal load was higher in the Infected animals compared to the Cy-Infected animals (Fig 7). However, despite the immunosuppression induced using cyclophosphamide, the percentage of CD8^+^ T lymphocytes in the lungs of the Cy-Infected group was higher compared to the Cy-Uninfected group (Fig 8A). Meanwhile, the CD4^+^ T lymphocytes were increased in the BAL of Cy-Infected group relative to the Cy-Uninfected group, while the percentage of CD8^+^ T lymphocytes decreased in the Cy-Infected group (Fig 8B). No differences were noted in the levels of blood T and B lymphocytes in all groups (S2B Fig).

**Fig 9:**
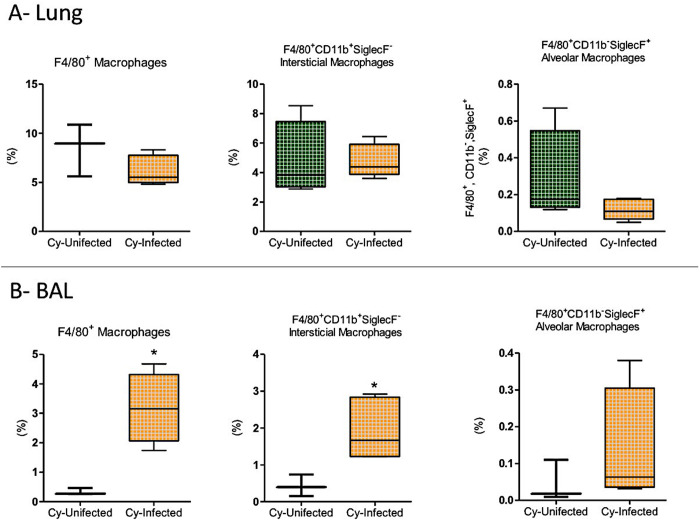
Macrophage populations in the lungs or bronchoalveolar lavage (BAL) of the mice immunosuppressed with cyclophosphamide (Cy) and then infected (Cy-Infected group) or not infected (Cy-Uninfected group) with *E. cuniculi* spores. **A)** Percentage of total macrophages (F4/80^+^), interstitial macrophages (F4/80^+^, CD11b^+^, and SiglecF^-^), and alveolar macrophages (F4/80^+^, CD11b^-^, and SiglecF^+^) in the lungs of mice. **B)** Percentage of total macrophages (F4/80^+^), interstitial macrophages (F4/80^+^, CD11b^+^, and SiglecF^-^), and alveolar macrophages (F4/80^+^, CD11b^-^, and SiglecF^+^) in BAL. The data presented are means ±standard errors of the mean (SEMs) (*p < 0.05, **p < 0.01, T-test).

The percentage of macrophages in the lung did not differ between the Cy-Infected and Cy-Uninfected groups, despite a trend toward reduced levels of alveolar macrophages (Fig 9A). However, in BAL, the percentage increase in the macrophages in the Cy-Infected group was evident, particularly due to increased levels of interstitial macrophages (F4/80^+^CD11b^+^SiglecF^-^) (Fig 9B). In the blood, increased levels of macrophages were detected in Infected (in percentual number) and Cy-Infected (in absolute number) (S3 Fig).

**Fig 10:**
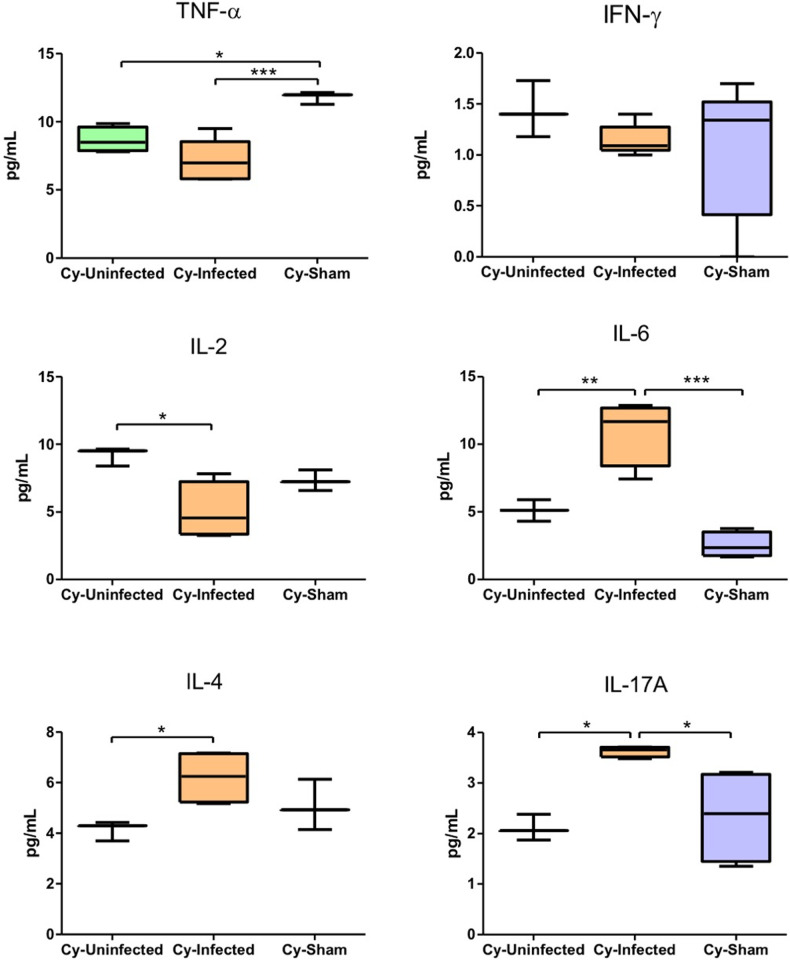
Plasma levels of TNF- α, IFN-γ, IL-6, IL-2, IL-17A, and IL-4 cytokines in the mice immunosuppressed with cyclophosphamide (Cy) and then infected (Cy-Infected group) or not infected (Cy-Uninfected group) with *E. cuniculi* spores and the uninfected and Cy-immunosuppressed mice that received the NaCl solution via the oropharyngeal route (Cy-Sham group). The data presented are means ±standard errors of the mean (SEMs) [*p < 0.05, **p < 0.01, ***p < 0.001; one-way analysis of variance (ANOVA) along with multiple comparisons and Tukey’s post-tests].

The cytokine profiles determined for the blood samples from the animals treated with Cy were quite different, with decreased levels of inflammatory cytokines TNF-α, IFN-γ, and IL-2 noted in the Cy-Infected group compared to the other groups, except for just IL-6, which was increased in this group (Fig 10). In addition, the anti-inflammatory cytokines IL-4 and IL-17A were increased in the Cy-Infected group (Fig 10). According to these results, the animals in this group presented a less inflamed profile.

## Discussion

The present study demonstrated the occurrence of interstitial pneumonia in all mice infected with *E. cuniculi* via the oropharyngeal route, thereby filling a gap in the epidemiological knowledge of this zoonosis, as this route of infection had not been demonstrated experimentally. The occurrence of pneumonia characterized by lymphoplasmacytic and occasionally mononuclear inflammatory infiltrate observed in the present study corroborated the results obtained in other previous studies on the intraperitoneal route of infection. In a previous study, for instance, *E. cuniculi* led to variable degrees of degeneration, necrosis, and desquamation of the bronchiolar epithelium and inflammatory infiltrates comprising mononuclear cells in the lung parenchyma of animals [13]. However, the pneumonia observed in the present study was more severe and the infection was further intense in non-immunosuppressed animals, which is different from the findings of the aforementioned authors who reported a greater number of lesions caused by *E. cuniculi* in immunosuppressed mice [13]. Cyclophosphamide is a non-specific cell cycle drug based on an alkylating agent derived from nitrogen mustard. It works by incorporating an alkyl group into the DNA, thereby preventing DNA synthesis and division. The immunosuppressive effects of cyclophosphamide (Cy) are attributed to its action on the DNA within the inflammatory cells, in addition to its suppressive effect on the bone marrow. The cytotoxic effect of this drug is due to the interaction of its alkylating metabolites with DNA. Treatment with Cy reportedly aggravated *E. cuniculi* infection, rendering it more severe, widespread, and with significantly increased lethality [9,14–16]. Contrary to the observations related to the intravenous infection of mice with *E. cuniculi* [17], in the present study, the fungal load was lower in the immunosuppressed group compared to the mice not treated with cyclophosphamide and then infected with *E. cuniculi*, which is quite intriguing. This difference may be attributed to the high population of CD8^+^ T lymphocytes detected in the lungs of infected groups of mice. The greater resistance to the pathogen observed in the Cy-Infected group was attributed to the higher percentage of CD8^+^ T lymphocytes detected in the lungs of the animals in this group. In addition, the Infected group mice exhibited a further inflamed cytokine profile, with increased levels of TNF, IFN, IL-2, and IL-6. These results demonstrated that infection via the oropharyngeal route facilitated the establishment of *E. cuniculi* infection in the lung tissues of mice with a consequent inflammatory response, resulting in predominant interstitial pneumonia. Previous studies have demonstrated that CD8^+^ T lymphocytes are the effector cells in encephalitozoonosis [18], and this could explain the lower fungal load noted in this group in the present study. Microsporidiosis plays a critical cytotoxic role, due to which this marked difference in the CD8 T lymphocyte population could explain the lower fungal load noted in the Cy-Infected group despite the immunosuppressive effect of Cy.

The lungs have two distinct populations of macrophages: alveolar macrophages (AM), which are in close contact with the type I and II epithelial cells of the alveoli, and interstitial macrophages (IM), which are located in the parenchyma between the microvascular endothelium and the alveolar epithelium. The functional phenotype of AM is modulated by the unique microenvironment of the lung, which comprises intimate contact with the epithelial cells, high oxygen tension, and exposure to surfactant-rich fluid, all of which are critical for tissue homeostasis, host defense, elimination of surfactant and cellular debris, pathogen recognition, initiation and resolution of lung inflammation, and repair of damaged tissue [19]. In the present study, an increase in AM populations (F4/80^+^CD11b^-^SiglecF^+^) was noted in the lungs and BAL of non-immunosuppressed infected animals, along with a greater fungal load and inflammatory infiltrate, indicating a further pronounced *Encephalitozoon* pneumonia. A milder pneumonia was observed in the mice deficient in AMs and infected with human metapneumovirus, with lower body weight loss, lung inflammation, and airway obstruction noted compared to the mice carrying AMs. On the other hand, pneumonia caused by syncytial virus was reported to be more severe in AM-deficient mice, suggesting that AM activity might vary depending on the causal agent [20]. The presence of AMs, reported in the present study, could be associated with further severe pneumonia in the encephalitozoonosis established via the oropharyngeal route.

AMs, as the first line of defense, initiate the innate immune response in the lung. However, 2 AM phenotypes may exist: the classically activated macrophages (AM–M1) and the alternatively activated macrophages (AM–M2). M1 macrophages respond to microbial factors and Th1 pro-inflammatory cytokines, thereby leading to glycolytic metabolism, which is associated with the release of inflammatory cytokines, increased bacterial killing, and recruitment of immune cells into the lung parenchyma and alveolus. M2 macrophages, on the other hand, are induced, by exposure to Th2 cytokines, leading to oxidative metabolism, which is associated with the release of anti-inflammatory cytokines, phagocytosis of apoptotic cells (efferocytosis), and collagen deposition, which contribute to the resolution of inflammation and repair of damaged tissues [19].

In the present study, it was hypothesized that the increased levels of AMs associated with the mixed cytokine profile of Th1 (IFN-γ, TNF-α, and IL-6) and Th2 (IL-4) observed in the serum samples from animals could be indicative of the concomitant presence of AMs from the M1 and M2 profiles in non-immunosuppressed animals. In a previous study, efferocytosis was observed to be favorable to *E. cuniculi* and induced the profile of M2 macrophages, thereby favoring the multiplication and dissemination of the pathogen [21], like the change in the profile of macrophages noted in the animals infected with *E. cuniculi*. A limitation of this study is the absence of longitudinal data and the lack of functional characterization of alveolar macrophage polarization (M1 vs. M2), which may restrict the depth of the immunological interpretation of our findings.

Interstitial macrophages (IMs) constitute the second line of defense in the lungs against invading pathogens. However, it is important to highlight that a dichotomy exists between IMs. Schyns et al. [22] demonstrated that the bronchial population of CD206^+^ IMs has a superior capacity to secrete immunoregulatory cytokines, including IL-10, consistent with the reported homeostatic functions of regulating inflammation and fibrosis. On the other hand, CD206^−^ MIs exhibited a typical antigen-presenting cell profile and were demonstrated to regulate the T cell-related processes. The increase in the levels of IMs in the lungs of infected mice observed in the present study and its association with a further severe infection might be suggestive of the adoption of an immunoregulatory profile, although further investigation is necessary for clarification. The levels of AM and IM macrophages were, on the other hand, not increased in the lungs of Cy-treated and infected mice, which demonstrated the resolution of infection during the period evaluated and also an increased population of CD8^+^ T lymphocytes. These inferences, however, must be understood better.

Previous studies have demonstrated that increased levels of Th1 cytokines in the serum of animals are associated with an increase in reactive oxygen and nitrogen products, which are important factors regarding resistance to *Encephalitozoon* infections in mice [18,23–25]. In the present study as well, a predominance of Th1 cytokines (TNF-α, IFN-γ, IL-2, and IL-6) was noted in the serum of infected mice.

IL-6 is a pleiotropic cytokine with several biological functions, including the regulation of the immune system through the production of other anti-inflammatory and pro-inflammatory cytokines [26]. Mice with diabetes mellitus (DM) exhibit greater susceptibility to encephalitozoonosis, which manifests as higher levels of IL-6 [18]. This was also demonstrated in the findings of the present study for animals not treated with Cy. In contrast, Cy-treated animals exhibited elevated IL-6 levels associated with the resolution of infection, suggesting an anti-inflammatory role of IL-6 and reiterating the ambiguity of this cytokine.

Th17 cytokines are categorized into six IL-17 subtypes, from A to F, which are produced by a broad spectrum of cell populations, including γδT, NKT, CD8^+^ T cells, neutrophils, microglia, and mast cells [4,27,28]. Other important sources of IL-17 include myeloid cells (e.g., in the kidneys and lungs) and Paneth cells in intestinal crypts [27]. IL-17A responses are required to control the infections caused by fungal pathogens, including *Aspergillus fumigatus, Pneumocystis carinii, Cryptococcus neoformans*, and *Candida albicans* [29–34]. IL-17C, on the other hand, induces lethal inflammation by exacerbating the secretion of pro-inflammatory cytokines, thereby contributing to the development of systemic fungal infection [32]. These paradoxical effects must be explored comprehensively. Under the conditions described in the present study, the increase in the serum IL-17 levels noted in the Cy-Infected group, which also had the lowest inflammation and fungal load, suggests that the role of this cytokine is consistent with the results obtained in previous studies on other fungal infections [29–34].

Among the limitations of this study, we can include the absence of analysis at additional time points of infection, which could have provided insights into the dynamics and progression of the infection. The sample size was not increased due to ethical concerns regarding the use of laboratory animals, with the experimental design following the principles of the 3Rs. Another limitation was the lack of phenotyping of alveolar macrophage subtypes, AM-M1 and AM-M2, which could have contributed to a better understanding of the interactions between innate immune cells and the adaptive immune response.”

Although often underestimated, oropharyngeal acquisition of encephalitozoonosis represents a relevant and emerging public health concern, particularly for immunocompromised individuals, elderly patients, and those in institutional or hospital settings. The potential for aspiration of contaminated secretions, combined with diagnostic challenges and limited clinical awareness, may lead to underdiagnosis and delayed treatment. Furthermore, the zoonotic and environmental distribution of *Encephalitozoon cuniculi* adds complexity to surveillance and prevention efforts. Recognizing this transmission route is essential to guide effective infection control measures, early diagnosis, and targeted interventions. Future research into the prevalence, transmission dynamics, and clinical outcomes associated with this route of infection may significantly improve public health strategies and patient care.

## Conclusion

The oropharyngeal route of infection promoted *E. cuniculi* pneumonia in the mice treated or not treated with cyclophosphamide, with a greater severity noted in non-immunosuppressed mice. Therefore, this model of infection could be established as a suitable model of interstitial pneumonia via aspiration, with the potential for the identification of the role of lung immune cells in infections caused by intracellular pathogens such as *E. cuniculi*.

## Supporting information

S1 Fig Hepatic granuloma (A and B) in the mice infected with *E. cuniculi* spores and then treated with cyclophosphamide (H&E). (TIFF)

S2 Fig The total percentage (%) of CD8^+^ and CD4^+^ T cell and B cell populations in the blood. A) The percentage of CD8^+^ and CD4^+^ T cell and B cell populations in the mice infected (Infected group) or not infected (Control group) with *E. cuniculi* spores. B) The percentage of CD8^+^ and CD4^+^ T cell and B cell populations in the mice immunosuppressed with cyclophosphamide (Cy) and then infected (Cy-Infected group) or not infected (Cy-Uninfected group) with *E. cuniculi* spores. The data presented are means ±standard errors of the mean (SEMs) (T-test).
(TIFF)

S3 Fig The absolute number or percentage (%) of macrophage populations in the blood. A) Mice infected (Infected group) or not infected (Control group) with *E. cuniculi* spores. B) Mice immunosuppressed with cyclophosphamide (Cy) and then infected (Cy-Infected group) or not infected (Cy-Uninfected group) with *E. cuniculi* spores. The data presented are means ±standard errors of the mean (SEMs) (*p < 0.05, ****p < 0.0001, T-test).
(TIFF)

## References

[1] Wadi L, Reinke AW. Evolution of microsporidia: An extremely successful group of eukaryotic intracellular parasites. PLoS Pathog. 2020;16(2):e1008276. doi: 10.1371/journal.ppat.1008276 32053705 PMC7017984

[2] Han B, Pan G, Weiss LM. Microsporidiosis in Humans. Clin Microbiol Rev. 2021;34(4):e0001020. doi: 10.1128/CMR.00010-20 34190570 PMC8404701

[3] Han B, Weiss LM. Microsporidia: Obligate Intracellular Pathogens Within the Fungal Kingdom. Microbiol Spectr. 2017;5(2):10.1128/microbiolspec.funk-0018–2016. doi: 10.1128/microbiolspec.FUNK-0018-2016 28944750 PMC5613672

[4] Moretto MM, Khan IA. Immune Response to Microsporidia. Exp Suppl. 2022;114:373–88. doi: 10.1007/978-3-030-93306-7_13 35544009

[5] Elango K, Mudgal M, Murthi S, Yella PR, Nagrecha S, Srinivasan V, et al. Trends in the Epidemiology and Outcomes of Pneumocystis Pneumonia among Human Immunodeficiency Virus (HIV) Hospitalizations. Int J Environ Res Public Health. 2022;19(5):2768. doi: 10.3390/ijerph19052768 35270461 PMC8910294

[6] Guarner J, Brandt ME. Histopathologic diagnosis of fungal infections in the 21st century. Clin Microbiol Rev. 2011;24(2):247–80. doi: 10.1128/CMR.00053-10 21482725 PMC3122495

[7] Kotkova M, Sak B, Kvetonova D, Kvac M. Latent microsporidiosis caused by Encephalitozoon cuniculi in immunocompetent hosts: a murine model demonstrating the ineffectiveness of the immune system and treatment with albendazole. PLoS One. 2013;8(4):e60941. doi: 10.1371/journal.pone.0060941 23593356 PMC3623998

[8] Kicia M, Szydłowicz M, Cebulski K, Jakuszko K, Piesiak P, Kowal A, et al. Symptomatic respiratory Encephalitozoon cuniculi infection in renal transplant recipients. Int J Infect Dis. 2019;79:21–5. doi: 10.1016/j.ijid.2018.10.016 30391325

[9] Anete Lallo M, Porta Miche Hirschfeld M. Encephalitozoonosis in pharmacologically immunosuppressed mice. Exp Parasitol. 2012;131(3):339–43. doi: 10.1016/j.exppara.2012.04.019 22626517

[10] Nielsen TB, Yan J, Luna B, Spellberg B. Murine Oropharyngeal Aspiration Model of Ventilator-associated and Hospital-acquired Bacterial Pneumonia. J Vis Exp. 2018;(136):57672. doi: 10.3791/57672 30010650 PMC6102004

[11] Moretto MM, Harrow DI, Hawley TS, Khan IA. Interleukin-12-producing CD103+ CD11b- CD8+ dendritic cells are responsible for eliciting gut intraepithelial lymphocyte response against Encephalitozoon cuniculi. Infect Immun. 2015;83(12):4719–30. doi: 10.1128/IAI.00820-15 26416905 PMC4645411

[12] Pfaffl MW. A new mathematical model for relative quantification in real-time RT-PCR. Nucleic Acids Res. 2001;29(9):e45. doi: 10.1093/nar/29.9.e45 11328886 PMC55695

[13] De Vooght V, Vanoirbeek JAJ, Haenen S, Verbeken E, Nemery B, Hoet PHM. Oropharyngeal aspiration: an alternative route for challenging in a mouse model of chemical-induced asthma. Toxicology. 2009;259(1–2):84–9. doi: 10.1016/j.tox.2009.02.007 19428947

[14] Francisco Neto A, Dell’Armelina Rocha PR, Perez EC, Xavier JG, Peres GB, Spadacci-Morena DD, et al. Diabetes mellitus increases the susceptibility to encephalitozoonosis in mice. PLoS One. 2017;12(11):e0186954. doi: 10.1371/journal.pone.0186954 29091912 PMC5665527

[15] Moysés CRS, Alvares-Saraiva AM, Perez EC, Spadacci-Morena DD, Vidôto da Costa LF, Xavier JG, et al. Mice with genetic and induced B-cell deficiency as a model for disseminated encephalitozoonosis. Comp Immunol Microbiol Infect Dis. 2022;81:101742. doi: 10.1016/j.cimid.2021.101742 35074660

[16] Hidifira AM, Alvares-Saraiva AM, Perez EC, Spadacci-Morena DD, de Araujo RS, Lallo MA. Increased susceptibility to encephalitozoonosis associated with mixed Th1/Th2 profile and M1/M2 profile in mice immunosuppressed with cyclophosphamide. Exp Parasitol. 2023;254:108606. doi: 10.1016/j.exppara.2023.108606 37666408

[17] Figuerêdo Moreira I, Marcelino Alvares-Saraiva A, Cristina Pérez E, Guilherme Xavier J, Denelle Spadacci-Morena D, Silva de Araújo R, et al. Opportunistic pneumonia caused by E. cuniculi in mice immunosuppressed with cyclophosphamide. Immunobiology. 2022;227(3):152194. doi: 10.1016/j.imbio.2022.152194 35278762

[18] Moretto MM, Weiss LM, Combe CL, Khan IA. IFN-gamma-producing dendritic cells are important for priming of gut intraepithelial lymphocyte response against intracellular parasitic infection. J Immunol. 2007;179(4):2485–92. doi: 10.4049/jimmunol.179.4.2485 17675510 PMC3109618

[19] Hu G, Christman JW. Editorial: Alveolar Macrophages in Lung Inflammation and Resolution. Front Immunol. 2019;10:2275. doi: 10.3389/fimmu.2019.02275 31616438 PMC6768960

[20] Kolli D, Gupta MR, Sbrana E, Velayutham TS, Chao H, Casola A, et al. Alveolar macrophages contribute to the pathogenesis of human metapneumovirus infection while protecting against respiratory syncytial virus infection. Am J Respir Cell Mol Biol. 2014;51(4):502–15. doi: 10.1165/rcmb.2013-0414OC 24749674 PMC4189482

[21] Dalboni LC, Alvares Saraiva AM, Konno FT de C, Perez EC, Codeceira JF, Spadacci-Morena DD, et al. Encephalitozoon cuniculi takes advantage of efferocytosis to evade the immune response. PLoS One. 2021;16(3):e0247658. doi: 10.1371/journal.pone.0247658 33667240 PMC7935246

[22] Schyns J, Bai Q, Ruscitti C, Radermecker C, De Schepper S, Chakarov S, et al. Non-classical tissue monocytes and two functionally distinct populations of interstitial macrophages populate the mouse lung. Nat Commun. 2019;10(1):3964. doi: 10.1038/s41467-019-11843-0 31481690 PMC6722135

[23] Franzen C, Müller A, Hartmann P, Salzberger B. Cell invasion and intracellular fate of Encephalitozoon cuniculi (Microsporidia). Parasitology. 2005;130(Pt 3):285–92. doi: 10.1017/s003118200400633x 15796011

[24] Langanke D, Alvares-Saraiva AM, Xavier JG, Spadacci-Morena DD, Peres GB, Rocha PRDA, et al. B-1 cells upregulate CD8 T lymphocytes and increase pro-inflammatory cytokines serum levels in oral encephalitozoonosis. Microbes Infect. 2018;20:196–204. doi: 10.1016/j.micinf.2017.11.004 29203270

[25] Han Y, Gao H, Xu J, Luo J, Han B, Bao J, et al. Innate and Adaptive Immune Responses Against Microsporidia Infection in Mammals. Front Microbiol. 2020;11:1468. doi: 10.3389/fmicb.2020.01468 32670257 PMC7332555

[26] da Costa LFV, Alvares-Saraiva AM, Dell’Armelina Rocha PR, Spadacci-Morena DD, Perez EC, Mariano M, et al. B-1 cell decreases susceptibility to encephalitozoonosis in mice. Immunobiology. 2017;222(2):218–27. doi: 10.1016/j.imbio.2016.09.018 27707513

[27] Khader SA, Gaffen SL, Kolls JK. Th17 cells at the crossroads of innate and adaptive immunity against infectious diseases at the mucosa. Mucosal Immunol. 2009;2(5):403–11. doi: 10.1038/mi.2009.100 19587639 PMC2811522

[28] Chiricozzi A, Guttman-Yassky E, Suárez-Fariñas M, Nograles KE, Tian S, Cardinale I, et al. Integrative responses to IL-17 and TNF-α in human keratinocytes account for key inflammatory pathogenic circuits in psoriasis. J Invest Dermatol. 2011;131(3):677–87. doi: 10.1038/jid.2010.340 21085185

[29] Rudner XL, Happel KI, Young EA, Shellito JE. Interleukin-23 (IL-23)-IL-17 cytokine axis in murine Pneumocystis carinii infection. Infect Immun. 2007;75(6):3055–61. doi: 10.1128/IAI.01329-06 17403873 PMC1932856

[30] Murdock BJ, Huffnagle GB, Olszewski MA, Osterholzer JJ. Interleukin-17A enhances host defense against cryptococcal lung infection through effects mediated by leukocyte recruitment, activation, and gamma interferon production. Infect Immun. 2014;82(3):937–48. doi: 10.1128/IAI.01477-13 24324191 PMC3957981

[31] Sparber F, LeibundGut-Landmann S. Interleukin 17-Mediated Host Defense against Candida albicans. Pathogens. 2015;4(3):606–19. doi: 10.3390/pathogens4030606 26274976 PMC4584276

[32] Huang J, Meng S, Hong S, Lin X, Jin W, Dong C. IL-17C is required for lethal inflammation during systemic fungal infection. Cell Mol Immunol. 2016;13(4):474–83. doi: 10.1038/cmi.2015.56 26166766 PMC4947823

[33] Lévy R, Okada S, Béziat V, Moriya K, Liu C, Chai LYA, et al. Genetic, immunological, and clinical features of patients with bacterial and fungal infections due to inherited IL-17RA deficiency. Proc Natl Acad Sci U S A. 2016;113(51):E8277–85. doi: 10.1073/pnas.1618300114 27930337 PMC5187691

[34] Guerra ES, Lee CK, Specht CA, Yadav B, Huang H, Akalin A, et al. Central Role of IL-23 and IL-17 Producing Eosinophils as Immunomodulatory Effector Cells in Acute Pulmonary Aspergillosis and Allergic Asthma. PLoS Pathog. 2017;13(1):e1006175. doi: 10.1371/journal.ppat.1006175 28095479 PMC5271415

