# Supplementary figures and images for: Interstitial pneumonia via the oropharyngeal route of infection with Encephalitozoon cuniculi
Source: PLoS Negl Trop Dis. 2025 Sep 8;19(9):e0012130. doi: 10.1371/journal.pntd.0012130 (PMC12435652; doi:10.1371/journal.pntd.0012130)

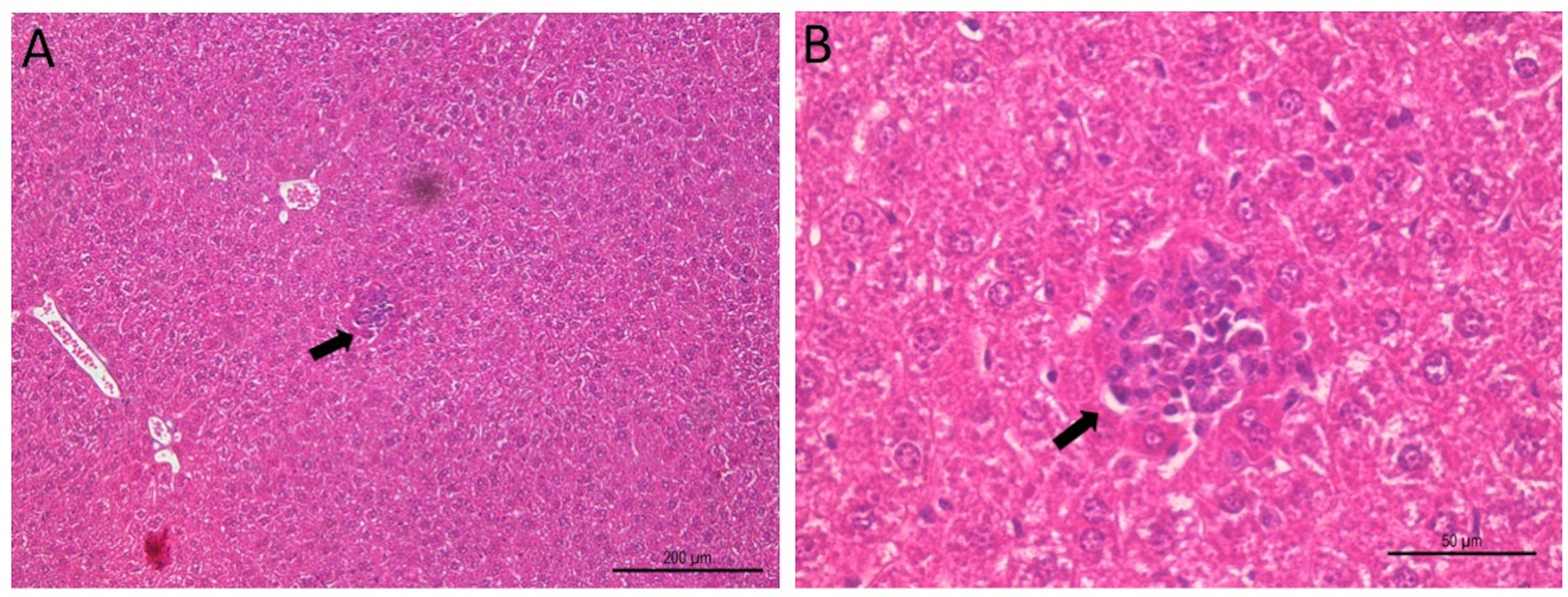

Supplement: S1 Fig — (TIFF) [file pntd.0012130.s001.tiff]

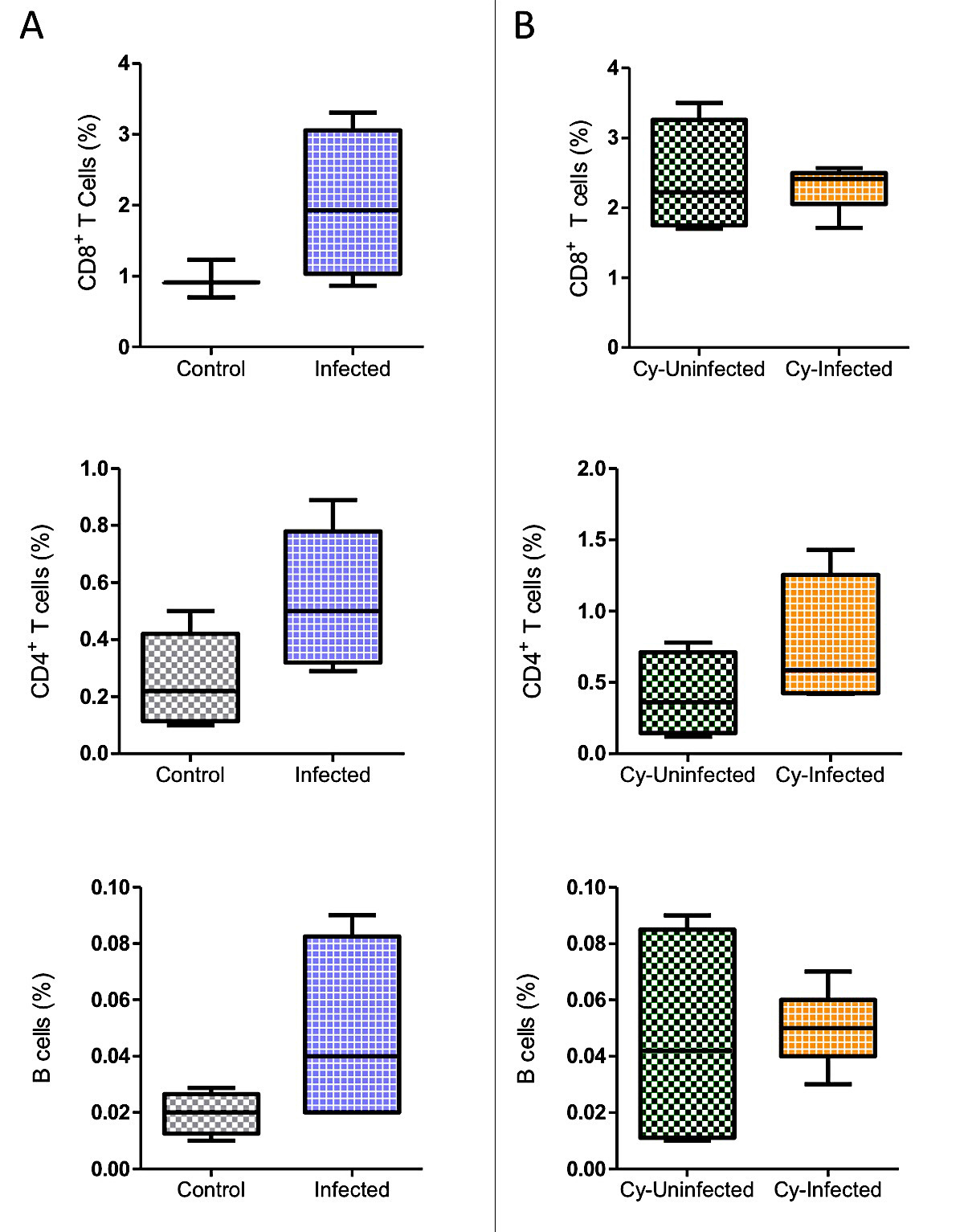

Supplement: S2 Fig — A) The percentage of CD8+ and CD4+ T cell and B cell populations in the mice infected (Infected group) or not infected (Control group) with E. cuniculi spores. B) The percentage of CD8+ and CD4+ T cell and B cell populations in the mice immunosuppressed with cyclophosphamide (Cy) and then infected (Cy-Infected group) or not infected (Cy-Uninfected group) with E. cuniculi spores. The data presented are means ±standard errors of the mean (SEMs) (T-test). (TIFF) [file pntd.0012130.s002.tiff]

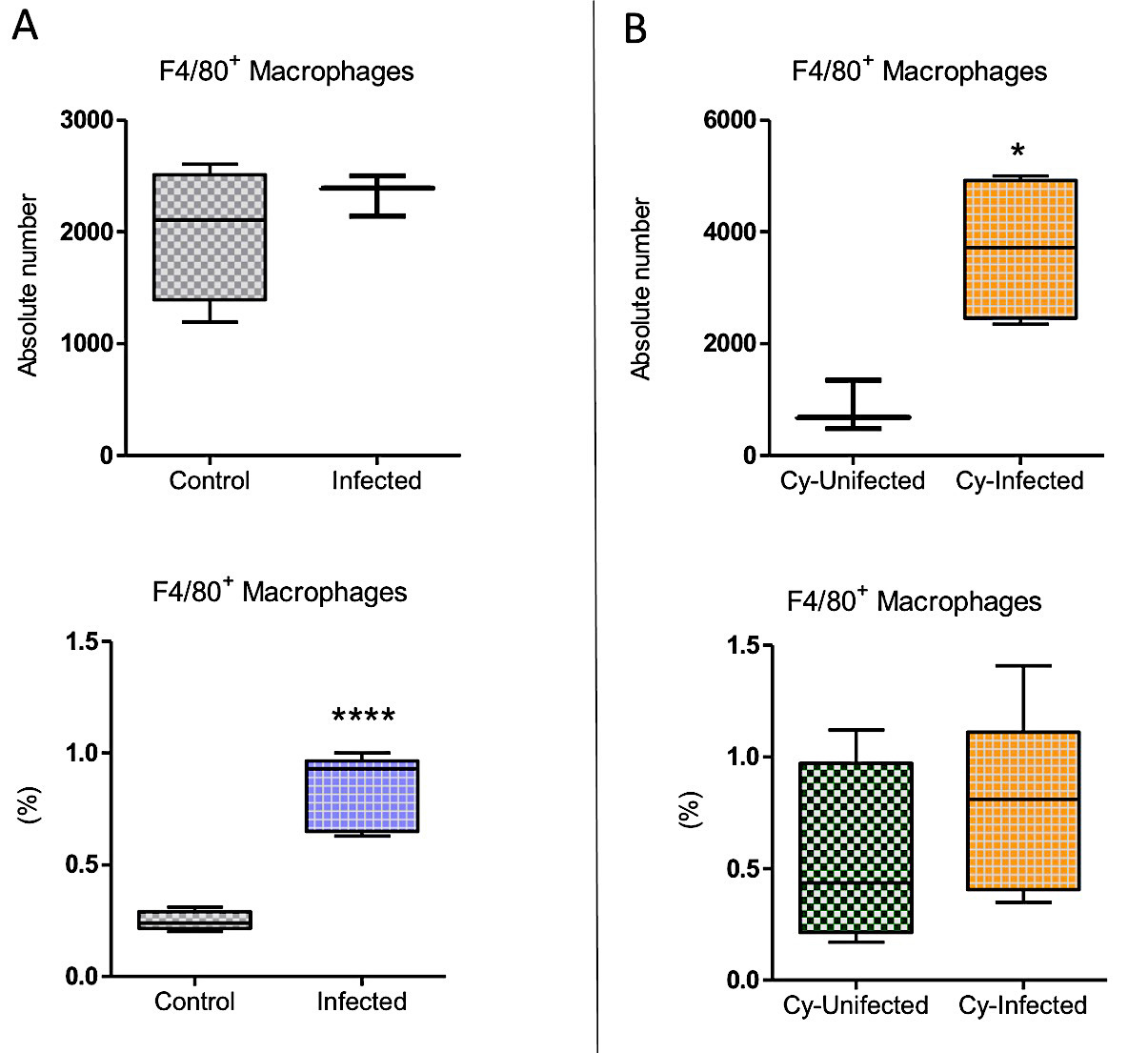

Supplement: S3 Fig — A) Mice infected (Infected group) or not infected (Control group) with E. cuniculi spores. B) Mice immunosuppressed with cyclophosphamide (Cy) and then infected (Cy-Infected group) or not infected (Cy-Uninfected group) with E. cuniculi spores. The data presented are means ±standard errors of the mean (SEMs) (*p < 0.05, ****p < 0.0001, T-test). (TIFF) [file pntd.0012130.s003.tiff]
